# Heat Stress Effects on Physiological and Blood Parameters, and Behavior in Early Fattening Stage of Beef Steers

**DOI:** 10.3390/ani13071130

**Published:** 2023-03-23

**Authors:** Won-Seob Kim, Jalil Ghassemi Nejad, Keun-Kyu Park, Hong-Gu Lee

**Affiliations:** Department of Animal Science and Technology, Sanghuh College of Life Sciences, Konkuk University, Seoul 05029, Republic of Korea

**Keywords:** heat stress, physiological parameters, blood parameters, behavior parameters, beef steers

## Abstract

**Simple Summary:**

This study explores the effects of heat stress on physiological, blood, and behavioral parameters in beef steers. We found that heat stress increases water intake, heart rate, rectal temperature, blood cortisol, heat shock protein 70, and time spent standing. However, there was no difference in intake parameters such as dry matter intake, blood glucose, and non-esterified fatty acids (NEFA). The findings indicated that physiological, blood, and behavioral parameters can be used as indicators of heat stress in beef steers and that heat resistance in beef steers is stronger than that of beef calves.

**Abstract:**

This study was conducted to investigate the effect of heat stress (HS) on physiological, blood, and behavioral parameters, according to the temperature–humidity index (THI), in beef steers. Twelve Korean native beef steers (342.7 ± 13.81 days old, body weight (BW) of 333.0 ± 18.53 kg) were used in this experiment. Beef steers were randomly distributed into three homogenized groups (four beef steers each) for 14 days, namely, threshold (THI = 64–71), mild–moderate (THI = 72–79), and severe (THI = 80–87). Feed and water intake were recorded daily. Physiological parameters, including heart rate and rectal temperature, and behavioral patterns (standing and lying down) were measured weekly. Blood was sampled every week to analyze hormones, heat shock protein (HSP) levels, metabolites, and hematological parameters. All data were analyzed using repeated-measures analysis. Beef steers exposed to severe THI had significantly increased (*p* < 0.001) water intake, heart rate, and rectal temperature compared to the threshold and mild–moderate THI beef steers. Additionally, increased blood cortisol (*p* < 0.001), HSP70 (*p* < 0.001), blood urea nitrogen (BUN) (*p* = 0.014), and time spent standing (*p* < 0.001) were observed in beef steers after exposure to severe THI compared to beef steers in the threshold and mild–moderate THI groups. However, dry matter intake, blood glucose, and non-esterified fatty acids were not different among the THI groups. In conclusion, heart rate, rectal temperature, blood cortisol, HSP70, BUN, and time spent standing were closely associated with severe HS conditions in beef steers. These phenomena indicated that beef steers exposed to HS modulated their behavior and blood parameters, as well as their physiological response, to maintain homeostasis.

## 1. Introduction

In many regions of the world, periods of heat stress (HS) are gradually increasing due to global warming [1]. Cattle experience HS when ambient temperature and humidity exceed their thermoneutral zone [2]. Heat stress can create multiple issues for cattle, such as increased metabolic disorders and decreased growth performance, immune function, and lactation [2,3]. Heat stress in beef cattle reduces nutrient intake, body weight (BW) gain, and meat quality, all of which can potentially cause large economic losses [4,5,6]. It is estimated that HS causes approximately USD 370 million in losses for the beef cattle industry [7].

The temperature–humidity index (THI) is an indicator of HS calculated using environmental factors such as air temperature and relative humidity [8]. It can be used to estimate cow cooling requirements to improve the production and effectiveness of management strategies during HS [8]. Previous studies reported reduced milk yield and dry matter intake (DMI) in addition to increased water intake and physiological parameters, including heart rate, rectal temperature, and skin temperature, in cows exposed to HS [8,9,10]. These external indicators are utilized by the THI to separate stress levels (threshold, mild, moderate, and severe) in dairy cows exposed to HS [8]. However, these previous studies focused on physiological parameters through exogenous reactions, especially productive ones. This does not reflect the response of internal performance, especially regarding energy metabolism, hormones, and blood metabolite. Furthermore, there is a relative paucity of research on HS in beef cattle, as production parameters in beef cattle are more difficult to ascertain than in dairy cows.

In our previous studies [3,4,5,11,12], we combined not only physiological indicators but also blood metabolites, heat shock protein (HSP) 70, behavior parameters, etc., to determine HS levels in beef calves. Blood metabolites, such as glucose and blood urea nitrogen (BUN) levels, changed during HS due to reduced DMI [3]. Heat shock protein 70 is accumulated immediately after cells are exposed to stressors, such as hypoxia, metabolic stress, and HS. This molecular chaperone aids in maintaining homeostasis and protecting cells from damage [13]. It also aids in using the HS parameters as indicators in tandem with HSP70 because HSP70 is the most sensitive among HSPs [14].

Behavioral patterns are also one of the important indicators concerning HS in beef cattle. Increased standing time allows cows to maximize the effective surface area for sensible and insensitive heat dissipation from the body surface, as shown in a previous study [15]. Heat resistance varies with age, and the period of 12–13 months is important for skeletal muscle and fat growth. There is, however, little information on how HS affects physiological, blood, and behavioral parameters in beef steers aged 12–13 months.

With these previous studies in beef calves, we hypothesized that different responses to stress parameters caused by HS would appear differently depending on the age of the beef cattle. Therefore, the objective of this study was to evaluate the responses of various stress parameters (such as physiological, blood, and behavioral) to HS conditions in beef steers. 

## 2. Materials and Methods

All the procedures involving animals were approved by the Institutional Animal Care and Use Committee (IACUC) of Konkuk University (Approval No: KU16054).

### 2.1. Animals, Experimental Design, and Diets

**Figure 1:**
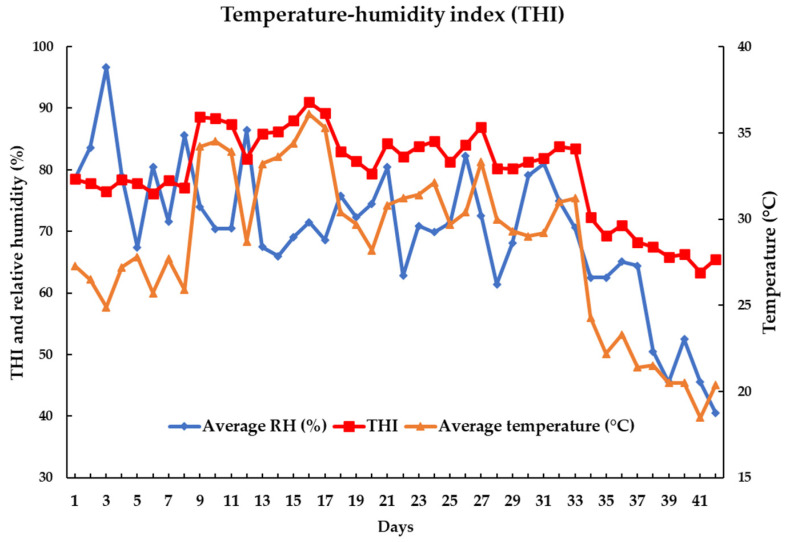
Changes in the temperature, relative humidity, and THI (temperature–humidity index) during the experimental period (42 days).

Twelve Korean native beef steers (342.7 ± 13.81 days old with BW of 333.0 ± 18.53 kg) were used in this experiment. The animals were randomly distributed into three homogenized groups (four beef steers each) and each group was exposed to HS under a natural environment, namely, threshold (THI = 64–71, day 29–42), mild–moderate (THI = 72–79, day 1–14), and severe (THI = 80–87, day 15–28). The total experiment lasted 14 days for each group. All the animals were kept in individual pens over the course of the experiment to calculate feed and water intake. The animals were subjected to an acclimatization period of 7 days in individual pens. Four sensors (SHT7x, Sensirion AG, Laubisruetistrasse 508,712 Staefa ZH, Switzerland) were used to record the air temperature and relative humidity (RH) at intervals of 1 s during the experiment. The experimental farm was covered with a roof and the animals were raised indoors; therefore, the animals were protected from rainfall, wind, and direct sunlight during the experiment. Only ambient temperature and RH affected the HS conditions during the total period. The following formula was used to determine the THI based on dry bulb temperature, according to a previous study: (T_db_, °C) and RH: THI = (1.8 × T_db_ + 32) − [(0.55 − 0.0055 × RH) × (1.8 × T_db_ − 26.8)] [16]. Over the course of the experiment, the ambient temperature, RH, and THI fluctuated (Figure 1).

The diet used in this experiment was total mixed ration (TMR), and the ingredients and chemical compositions of the feed are shown in Table 1. The feed was provided twice a day at 0900 and 1700 h and the water was provided four times a day at 0900, 1300, 1700, and 2100 h. Every morning (0900 h) after the offering, the residues of both feed and water were calculated.

### 2.2. Chemical Analysis in Diets

According to AOAC [17], the following measurements were analyzed: dry matter (DM; method 930.15), crude protein (CP; method 984.13), acid detergent fiber (ADF; method 973.18), ether extract (EE; method 920.39), and ash (method 942.05). The method used by Van Soest, et al. [18] was used to analyze the neutral detergent fiber (NDF) content. Coupled plasma spectroscopy (method 945.46) was used to inductively estimate the Ca and P levels [17]. Ash content was determined by incineration at 550 °C overnight in a muffle furnace, and dry matter was assessed by drying ground diets in a vacuum oven at 100 °C overnight (KMF-500, Lab Corporation, Seoul, Republic of Korea). The Kjeltec™ System (Kjeltec™ 2400, FOSS, Denmark) was used to assess the total nitrogen in the feed to determine the CP contents. The final CP content was calculated as nitrogen × 6.25. The ether extraction system (ANKOMXT15 Extractor, ANKOM Technology, Macedon, NY, USA) was used to measure the amount of ether extract.

### 2.3. Physiological Parameters under Heat Stress

Heart rate and rectal temperature were measured every week (day 7 and day 14 in each period) at 1400 h. A large clinical animal thermometer (TES-1300 Thermometer; E&E PROCESS Instrument Co., Vaughan, ON, Canada) was used to measure the rectal temperature. It was inserted into the rectum of beef steers to a depth of 3 cm and kept in contact with the mucosa for 1 min. A stethoscope (TS-DIA01002; Tenso Medical Instrument Co., 238 Zhongshan, Ningbo, China) was placed directly onto the left thoracic region under one of the auscultation foci for 1 min in order to measure heart rate, which is expressed in beats per minute (BPM).

### 2.4. Blood and Behavior Parameters under Heat Stress

Every week (day 7 and day 14 in each period) at 1100 h, blood samples from the jugular veins of beef steers were taken for serum extraction (20 mL; Becton-Dickinson, Belliver Industrial Estate, PL6 7BP, Plymouth, UK) and hematology (4 mL; Becton-Dickinson, Franklin Lakes, NJ, USA) analysis serum samples were obtained from blood samples after centrifugation at 2700× *g* for 15 min at 4 °C. Serum was transferred to a 1.5 mL tube (Eppendorf AG, Hamburg, Germany) and kept at −80 °C until analysis.

Using a commercial bovine ELISA test kit, the levels of serum cortisol and HSP70 were examined (Life Diagnostics, Inc, West Chester, PA, USA; Endocrine Technologies, Inc, Newark, CA, USA). JW Medical (Seoul, Republic of Korea) provided the analytical reagents to measure the levels of glucose, glutamic-oxaloacetic transaminase (GOT), and glutamic-pyruvate transaminase (GPT). Wako Pure Chemical (Osaka, Japan) provided the analytical reagents to measure the levels of non-esterified fatty acids (NEFA), BUN, triglycerides (TG), creatine (CREA), high-density lipoproteins (HDL), low-density lipoproteins (LDL), calcium (CA), inorganic phosphorus (IP), and magnesium (MG). An automated chemical analyzer (Hitachi 7180, Tokyo, Japan) was used to measure all metabolites. Using a VetScan HM2 (Diamond Diagnostics, Abaxix Inc., Holliston, MA, USA), whole blood was analyzed for white blood cell (WBC) and platelet counts as hematological traits. 

Four cameras (SNV-7080R, Hanwha Techwin, Changwon, Republic of Korea) were used to record standing and lying down behavioral patterns. Every week, between the hours of 0900 and 1900, the times spent standing or lying down were recorded (600 min).

### 2.5. Statistical Analysis

All data were analyzed using repeated-measures analysis and the GLM procedure in SAS version 9.4 (SAS Institute Inc., Cary, NC, USA). The model used was as follows:

Y*_ijk_* = µ + α*_i_* + β*_j_* + γ(α)*_ik_* + ε*_ijk_*

where Y*_ijk_* is the observation of beef steer *k* at sampling time *j* for a given treatment *i*, µ is the overall mean, α*_i_* is the fixed effect of treatment *i* (THI), β*_j_* is the fixed effect of sampling time *j* (every week), γ(α)*_ik_* is the random effect of beef steer *k* nested in treatment *i*, and ε*_ijk_* is the residual effect. The model included a random effect for beef steer identification. The subject of the repeated statement was the effect of beef steers. For mean comparisons, Tukey’s honest significant difference (HSD) test was used. Covariance structures (autoregressive order 1, unstructured and compound symmetry) for the repeated-measures model were tested. The structure that best fits the model was chosen based on the smallest value of Schwarz’s Bayesian information criterion. The first day of sampling in each THI group was included as a covariate to adjust the means. The covariate factor was included in the model when appropriate, but was removed from the model when it was insignificant.

Although a study with a larger number of animals would provide more accurate physiological patterns, we were unable to increase the number of animals in our study due to limited resources. Instead, a post hoc power analysis was conducted (G*Power, version 3.1.9.7, University of Dusseldorf, Dusseldorf, Germany) to ensure that our analysis had adequate statistical power to detect differences among the groups and confirmed that the power of analysis (1 − β) was 0.95. The least square means of the data are presented, along with standard errors. Differences were considered statistically significant if the *p*-value was less than 0.05. Means with *p*-values between 0.05 and 0.10 reflected a tendency to differ significantly.

## 3. Results

### 3.1. Intake and Physiological Parameters

Beef steers exposed to severe HS (THI 80–87) had significantly increased (*p* = 0.001) water intake compared to the threshold and mild–moderate HS (THI 64–79) groups (Table 2). However, DMI did not differ (*p* = 0.072) significantly between the groups (Table 2).

Heart rate and rectal temperature increased (*p* < 0.001) with severe THI levels compared to threshold and mild–moderate THI levels (Table 2).

### 3.2. Blood Parameters

Increased blood cortisol and HSP70 levels were observed after exposure to severe THI compared to threshold and mild–moderate THI (*p* < 0.001) (Table 3).

Beef steers exposed to severe HS (THI 80–87) had significantly increased (*p* = 0.007) BUN levels compared to those of the threshold and mild–moderate HS (THI 64–79) groups (Table 4). However, blood glucose, NEFA, GOT, GPT, TG, CREA, HDL, LDL, CA, IP, MG, WBC, and platelet levels were not significantly different between the groups (*p* > 0.05) (Table 4).

### 3.3. Behavior Parameters

Beef steers exposed to severe HS (THI 80–87) had significantly increased (*p* < 0.001) time spent standing compared to the threshold HS (THI 64–71) group (Table 5). Contrarily, time spent lying down was decreased (*p* < 0.001) in the severe HS group (THI 80–87) (Table 5). 

## 4. Discussion

Livestock are exposed to a variety of stressors that affect growth performance, milk production, immune function, welfare, and health. Heat stress is one of the most important factors affecting growth performance and economic losses in livestock [2,7]. Regarding this, average ambient temperatures have continuously increased during the summer season due to global warming, further aggravating damage to livestock [1,7]. HS causes behavioral and metabolic changes in cattle that result in lower productivity and profitability [3,5]. Many previous studies have demonstrated that the physiological responses of cows in the body, such as intake, heart rate, and rectal temperature, change to maintain homeostasis after exposure to HS [19,20]. However, there has been little research on HS because direct production indicators, such as milk production, are generally more difficult to identify in beef cattle than in dairy cows. Our previous studies revealed that HS affects various stress parameters in growing-stage beef calves (5–6 months old) [3,4,5,11,12,13]. However, as beef cattle grow and their bodies’ metabolic mechanisms change, other HS responses can be observed in beef steers (12–13 months old). The results of this study can therefore improve our understanding of how HS can impair the ability of beef cattle to cope with these outbreaks.

Productivity, described by indicators such as average daily gain and milk production, is used as a major indicator for measuring stress status in the ruminant industry [2,3,21]. In general, HS affects productivity in cattle because DMI is reduced [3,5]. Following a reduction in DMI, increased water intake is the first noticeable signal of HS, and presumably began as an evolutionary strategy to reduce the heat increment associated with feeding [6]. Our previous studies reported that HS affects DMI reduction in growing beef calves [3,5]. Heat exposure can disturb the counterbalance between metabolic heat production and dissipation, altering the cattle’s metabolism [3]. In the current study, severe THI increased water intake in beef steers. Drinking more water is the most important phenomenon for beef cattle, and it has a high specificity factor for promoting heat dissipation [22]. Previous studies have found links between water intake and environmental temperature, and between water intake and rumen temperature [22,23,24]. Ruminal microbial composition is altered when animals are subjected to HS, which leads to metabolic diseases [25]. Water intake requirements increased to control rumen status during HS in beef cattle. However, previous studies indicated that water intake was not affected by HS in growing beef bulls [3,26]. According to this phenomenon, rumen microbial composition and development differ depending on the growth period and occur in different mechanisms for preventing HS in beef cattle based on age. There is no significant difference in DMI during HS exposure in the current study. Additionally, blood glucose and NEFA levels related to feed intake indicators were not changed. This is contrary to the results of previous studies [3,6,21]. In general, feed intake is reduced in HS, and the body supplies energy sources through mechanisms such as gluconeogenesis, thereby restoring decreased glucose and controlling NEFA levels [3,6] in beef calves and cows. Presumably, HS has no significant effect on feed intake through water intake regulation, in addition to various homeostasis mechanisms in the body, during the early fattening stage of beef steers.

Physiological parameters, including heart rate and rectal temperature, are the most commonly used indicators for cattle exposed to HS [8,12]. Heart rate and rectal temperature may have responded to homeostatic disturbances, resulting in a range of physiological changes under HS. In the current study, severe THI increased heart rate and rectal temperature in beef steers. Previous studies reported that cattle exposed to sudden HS without acclimation for the first time exhibited increased heart rate and rectal temperature, which is consistent with a stress response [12,20,27]. This finding is in agreement with our previous study, which showed that heart rate and rectal temperature increased during short-term exposure to HS for 4 days in growing beef calves [4]. Heart rate and rectal temperature are indicators of heat balance in the body and can be used to assess the adverse effects of HS on performance and productivity in dairy cows [2] and beef calves [4,5,12]. Heart rate and core body temperature are nearly constant under normal conditions, making them sensitive indicators of physiological responses to HS in bovines, especially in beef steers.

External stress indicators such as feed intake, water intake, heart rate, and rectal temperature are used to separate stress levels in the THI chart in dairy cows exposed to HS [8]. However, these do not reflect the response of internal responses such as blood hormones or metabolites. In the current study, blood parameters, including cortisol, HSP70, and metabolites, were also analyzed together to find more accurate stress responses and to understand how HS affects the body in beef steers. Increased blood cortisol levels are closely associated with abnormal animal behaviors such as anxiety and sensitivity [28]. The hypothalamic–pituitary–adrenal and sympathetic–adrenal–medullary axes are enabled to conserve homeostasis in response to HS by stimulating blood hormone levels after exposure to HS in animals [29]. Blood cortisol acts as a stimulus to the immune system during acute stress, and chronic stress is associated with immune suppression [30]. HSPs are accumulated immediately after cells are exposed to stressors, such as hypoxia and HS. These molecular chaperones maintain homeostasis and protect cells from HS damage [13]. HSP70 is one of the most sensitive proteins to HS among HSPs. Previous studies have reported that HSP70 expression from cells, tissues, blood, and hair follicles was increased after sudden exposure to HS in beef cattle [12,13,31]. In the current study, blood cortisol and HSP70 levels were increased in severe THI. These results were consistent with the results of previous studies [3,4] and showed no difference by growth stage, unlike the results of DMI. BUN is generally produced in the liver from the rumen (inefficient rumen ammonia incorporation into microbial proteins) and hepatic deamination of amino acids [32]. In the current study, BUN levels were significantly elevated for the severe THI group; however, it was unclear whether this increase in BUN levels was generated from skeletal muscle breakdown or excess rumen ammonia production. This may be presented as a result of HS-induced exposure through protein metabolism designed to activate protein utilization as an energy source. Blood GOT and GPT levels are liver damage markers and have been reported to signal impaired liver function during HS [33]. There was no difference in GOT and GPT levels among the groups in the current study. Contrarily, in our previous study, HS affected blood GOT and GPT levels and liver function in growing beef calves [3,4]. These results are expected, probably because HS did not significantly affect DMI in early fattening-stage beef steers. Taken together, blood parameters including cortisol, HSP70, and BUN levels are closely associated with HS and may be used as sensitive indicators for determining stress levels in beef steers.

During the severe THI period, behavior patterns, including times spent standing and lying down, were changed in beef steers. Standing and lying down are behavioral indicators that are subject to change during HS. Previous studies have reported that behavioral patterns change in animals in response to HS [34,35]. Additionally, time spent standing increased and time spent lying down decreased when growing beef calves were exposed to HS in our previous studies [3,4]. More time spent standing may allow cattle to maximize effective surface area for sensible and insensible heat dissipation from body surfaces [15]. Lying down reduces heat from a warm surface and increases the efficiency of respiration in ruminants [15]. Previous studies have suggested that time spent lying down increases and standing time decreases when cattle are provided with shade [36,37]. Therefore, beef steer behaviors, including standing and lying down, are important eye-tracking indicators during HS.

## 5. Conclusions

This study used a variety of markers to stimulate the effect of short-term heat stress in Korean native beef steers, including intake, physiological and blood parameters, and behavioral patterns. In conclusion, it was found that severe heat stress conditions were closely correlated with water intake, heart rate, rectal temperature, blood parameters (cortisol, HSP70, and blood urea nitrogen), and behavioral patterns (standing and lying down). These phenomena indicated that beef steers exposed to heat stress modulated their behavioral patterns and blood parameters, as well as their physiological responses, to maintain homeostasis. The results of the present study further improve our understanding of the responses of beef steers to sudden heat stress exposure.

## Figures and Tables

**Table 1:** Diet ingredients and chemical compositions.

Ingredients, % of DM	
Molasses	2.06
Corn, cracked	14.64
Brewer’s grain	13.94
Almond hull	9.47
Corn gluten	26.57
Cotton seed	3.78
Alfalfa, hay	12.33
Klein grass, hay	8.95
Annual rye, straw	6.26
Limestone	1.34
Salt	0.39
Vitamin and mineral premix ^1^	0.27
Total	100
Chemical composition ^2^	
DM, %	64.60
CP, % of DM	14.40
NDF, % of DM	38.60
ADF, % of DM	21.50
EE, % of DM	3.40
Crude ash, % of DM	8.40
Ca, % of DM	1.10
P, % of DM	0.50

^1^ The premix contained (% as-is basis): trace mineral mix, 0.86; MgO (56% Mg), 8.0; NaCl, 6.4; vitamin A, D, E premix, 37.2; selenium premix, 0.07; dry corn distillers grains with solubles, 45.7%; Ca, 12.1%; P, 0.41%; Mg, 4.59%; K, 0.44%; S, 0.39%; Se, 6.91 mg/kg; Cu, 383 mg/kg; Zn, 884 mg/kg; Fe, 216 mg/kg, vitamin A, 300,000 IU/kg, vitamin D, 85,000 IU/kg, vitamin E, 2300 IU/kg. ^2^ DM = dry matter, CP = crude protein, NDF = neutral detergent fiber, ADF = acid detergent fiber, EE = ether extract, Ca = calcium, P = phosphorus.

**Table 2:** Effect of heat stress level on dry matter intake, water intake, and physiological parameters in Korean native beef steers.

Items ^1^	THI ^2^			SEM	*p*-Value
	Threshold (64–71)	Mild–Moderate (72–79)	Severe (80–87)		
DMI (kg/day)	9.94	9.59	9.22	0.206	0.072
Water intake (L/day)	45.89 ^b^	45.82 ^b^	63.19 ^a^	3.265	0.001
Heart rate (BPM)	62.75 ^b^	65.75 ^b^	72.13 ^a^	1.134	<0.001
Rectal temperature (°C)	38.65 ^c^	38.86 ^b^	39.18 ^a^	0.046	<0.001

^1^ DMI = dry matter intake, BPM = beats per minute. ^2^ THI = temperature–humidity index. ^a–c^ Mean values with different letters differ significantly (*p* < 0.05).

**Table 3:** Effect of heat stress level on blood cortisol and heat shock protein 70 levels in Korean native beef steers.

Items ^1^	THI ^2^			SEM	*p*-Value
	Threshold (64–71)	Mild–Moderate (72–79)	Severe (80–87)		
Cortisol (ng/mL)	1.91 ^c^	5.13 ^b^	9.87 ^a^	0.762	<0.001
HSP70 (ng/mL)	25.39 ^b^	36.56 ^ab^	47.87 ^a^	3.175	<0.001

^1^ HSP70 = heat shock protein 70. ^2^ THI = temperature–humidity index. ^a–c^ Mean values with different letters differ significantly (*p* < 0.05).

**Table 4:** Effect of heat stress level on blood metabolites and hematological parameters in Korean native beef steers.

**Items ^1^**	**THI ^2^**			**SEM**	***p*** **-Value**
	Threshold (64–71)	Mild–Moderate (72–79)	Severe (80–87)		
Glucose (mg/dL)	84.75	86.00	85.86	2.021	0.891
NEFA (µEq/L)	83.13	100.81	94.81	6.668	0.187
BUN (mg/dL)	15.50 ^b^	16.25 ^ab^	18.25 ^a^	0.562	0.007
GOT (U/dL)	83.13	76.50	75.63	4.811	0.495
GPT (U/dL)	25.38	25.75	26.75	1.020	0.622
TG (mg/dL)	18.25	16.75	22.30	2.190	0.082
CREA (mg/dL)	1.03	1.03	1.02	0.023	0.914
HDL (mg/dL)	197.25	177.88	186.00	6.990	0.169
LDL (mg/dL)	29.00	25.50	29.50	2.138	0.371
CA (mg/dL)	10.55	10.59	10.81	0.089	0.100
IP (mg/dL)	9.30	9.19	9.50	0.148	0.337
MG (mg/dL)	3.08	3.23	3.13	0.057	0.190
WBC (k/µL)	12.85	10.28	8.82	3.425	0.102
Platelet (k/µL)	308.75	431.15	437.75	81.124	0.118

^1^ NEFA = non-esterified fatty acids, BUN = blood urea nitrogen, GOT = glutamic-oxaloacetic transaminase, GPT = glutamic-pyruvic transaminase, TG = triglycerides, CREA = creatine, HDL = high-density lipoproteins, LDL = low-density lipoproteins, CA = calcium, IP = inorganic phosphorus, MG = magnesium, WBC = white blood cells. ^2^ THI = temperature–humidity index. ^a,b^ Mean values with different letters differ significantly (*p* < 0.05).

**Table 5:** Effect of heat stress level on behavioral patterns in Korean native beef steers.

Items	THI ^1^			SEM	*p*-Value
	Threshold (64–71)	Mild–Moderate (72–79)	Severe (80–87)		
Standing time (min/day)	329.38 ^b^	374.38 ^a^	396.25 ^a^	9.348	<0.001
Lying time (min/day)	270.63 ^a^	225.63 ^b^	203.75 ^b^	9.348	<0.001

^1^ THI = temperature–humidity index. ^a,b^ Mean values with different letters differ significantly (*p* < 0.05).

## Data Availability

The data presented in this study are available in this paper.
